# Prognostic impact of rejection and chronicity index on long-term graft outcomes in pediatric kidney transplant recipients

**DOI:** 10.3389/fimmu.2026.1737410

**Published:** 2026-03-20

**Authors:** Jianming Li, Wenyu Xie, Yan Wang, Zicong Li, Bowen Xu, Qian Fu, Chenglin Wu, Ronghai Deng, Xiaojun Su, Xuntao He, Liuting Ling, Longshan Liu, Huanxi Zhang, Jun Li, Changxi Wang

**Affiliations:** 1 Organ Transplant Center, The First Affiliated Hospital, Sun Yat-sen University, Guangzhou, China; 2 Department of Medical Ultrasonics, Institute of Diagnostic and Interventional Ultrasound, The First Affiliated Hospital of Sun Yat-Sen University, Guangzhou, China

**Keywords:** activity index, banff classification, chronicity index, graft function, pediatric kidney transplantation, rejection, risk factors

## Abstract

**Background:**

Acute rejection remains a major complication in pediatric kidney transplantation. The Banff Activity Index (AI) and Chronicity Index (CI) have recently been proposed as quantitative composites of histological lesions that represent the extent of active and chronic injury, respectively. Emerging evidence suggests that these indices provide additional value for prognostic assessment. However, their clinical significance in pediatric recipients remains unclear.

**Methods:**

This single-center retrospective study included 535 pediatric kidney transplant recipients who underwent transplantation between 2015 and 2025. AI and CI were calculated according to Banff lesion scores. Logistic regression was used to identify risk factors for rejection, and graft functional outcomes were evaluated using Kaplan–Meier analysis and longitudinal estimated glomerular filtration rate (eGFR) trajectories across rejection phenotypes and AI/CI categories.

**Results:**

Among 535 recipients, 98 (18.3%) experienced 126 rejection episodes. Independent risk factors for rejection included HLA-DR mismatch (OR 1.60, 95% CI 1.09–2.43, p = 0.021), previous transplantation (OR 3.05, 95% CI 1.03–8.53, p = 0.036), preformed donor-specific antibodies (pfDSA) (OR 3.11, 95% CI 1.08–8.31, p = 0.027), and recipient aged 9–15 years (OR 2.05, 95% CI 1.30–3.29, p = 0.002). Both AI and CI scores varied significantly across rejection phenotypes, with mixed rejection showing the highest levels of histological activity and chronicity (AI: p < 0.001; CI: p = 0.017). Higher AI scores were associated with a stepwise decline in eGFR at diagnosis (p = 0.001), but this difference was no longer significant during long-term follow-up. In contrast, high CI (≥ 4) was linked to lower eGFR at 3 years post-rejection compared with low CI (< 4) (35.9 vs. 62.8 mL/min/1.73 m²; p = 0.016). High CI (≥ 4) at the first biopsy was independently associated with donation after circulatory death (DCD) (OR 3.95, p = 0.04) and biopsy performed ≥ 3 years post-transplantation (OR 3.80, p = 0.05).

**Conclusion:**

Acute rejection remains significantly associated with adverse long-term graft outcomes in pediatric kidney transplantation. CI ≥ 4 was associated with long-term functional decline, whereas AI primarily reflected short-term functional impairment.

## Introduction

Over recent decades, pediatric kidney transplantation has demonstrated excellent short-term outcomes (1). However, despite favorable short-term outcomes, the long-term prognosis remains suboptimal, with graft survival decreasing to approximately 60% at 10 years (2–4). Among the various post-transplant complications, rejection stands out as a major determinant of late graft dysfunction and graft loss (3–5). The incidence of acute rejection remains higher in pediatric kidney transplant recipients, with 1-year rates of approximately 10–18% compared with about 7% in adults (6–9). Beyond its impact on graft failure and mortality, acute rejection also increases post-transplant healthcare utilization and costs (10–12).

Although risk factors for acute rejection have been extensively investigated in adult kidney transplant recipients, data on pediatric populations remain limited (13, 14). Unlike adults, pediatric kidney transplant recipients have distinct immunological and clinical characteristics. In terms of immunology, pediatric recipients exhibit an immature adaptive immune system with reduced effector T-cell activity, lower costimulatory signaling, and diminished antigen-presenting capacity. Conversely, they exhibit innate-like responses that allow rapid Toll-like receptor (TLR)–mediated activation and cytokine release, shaping distinctive alloresponses (15–17). In terms of clinical characteristics, pediatric recipients are still undergoing physical and psychological maturation, which affects drug pharmacokinetics, dosing, and treatment adherence (18–20). Therefore, acute rejection in pediatric kidney transplant recipients may be influenced by unique risk factors that warrant further investigation.

Failure to detect or adequately treat acute rejection in its early phase frequently results in progressive chronic allograft injury, characterized by transplant glomerulopathy (TG), arterial intimal fibrosis, and interstitial fibrosis/tubular atrophy (IF/TA). The Banff classification remains the international standard for histopathological diagnosis and grading of rejection, and the 2019 update delineates three major subtypes of antibody-mediated rejection (ABMR): active, chronic active, and chronic (inactive), based on microvascular inflammation, evidence of antibody–endothelium interaction, and chronic structural changes (21). Although these refinements have enhanced diagnostic precision and prognostic stratification, they have also increased complexity and interobserver variability. In recognition of the heterogeneity within current rejection subtypes, the Banff Activity and Chronicity Indices Working Group proposed to complement these categories with graded activity and chronicity indices (AI and CI) to provide a more continuous and quantitative assessment of rejection severity (22). The AI is derived from active Banff lesions (i, t, v, g, ptc, and C4d), reflecting the inflammatory burden, whereas the CI integrates chronic Banff lesions (ci, ct, cv, and cg×2), indicating chronic structural injury. This approach acknowledges that rejection represents a continuum of disease activity and chronic damage, rather than discrete categories, and provides greater granularity for both prognosis and therapeutic decision-making. A recent study demonstrated that higher CI scores are strongly associated with adverse graft outcomes, with CI ≥ 4 identifying high-risk patients more accurately than AI (23). Similarly, AI provides complementary prognostic informations (22). Importantly, both indices can be utilized either independently of or in conjunction with rejection subtypes, thereby simplifying biopsy reporting, improving risk stratification, and potentially guiding individualized interventions (22). However, pediatric-specific validation remains limited.

In this retrospective study, we aimed to identify risk factors for rejection and evaluate the prognostic value of the Banff activity and chronicity indices in pediatric kidney transplant recipients.

## Materials and methods

### Study population

We retrospectively analyzed 551 pediatric kidney transplant recipients who underwent kidney transplantation at the Organ Transplant Center of the First Affiliated Hospital of Sun Yat-sen University between January 2015 and March 2025. Recipients who experienced early graft loss or were lost to follow-up were excluded. After excluding 16 cases, a total of 535 pediatric kidney transplant recipients were included in the final analysis.

### Induction and maintenance immunosuppression

All recipients received induction therapy consisting of high-dose corticosteroids (10 mg/kg) combined with either anti-thymocyte globulin or anti-CD25 monoclonal antibodies, according to immunological risk and institutional protocol. Maintenance immunosuppression primarily consisted of a calcineurin inhibitor (tacrolimus or cyclosporine), mycophenolate mofetil (MMF) or enteric-coated mycophenolate sodium (EC-MPS), and corticosteroids. In clinically stable recipients, steroid tapering or withdrawal was considered within 3–12 months post-transplantation. Immunosuppressive regimens were adjusted in cases of specific complications (e.g., BK or CMV infection), including calcineurin inhibitor modification or addition of mTOR inhibitors as clinically indicated.

### Definition and diagnosis of rejection episodes

Rejection episodes were categorized as biopsy-proven rejection and clinically diagnosed acute rejection. Biopsy-proven rejection refers to the classification of rejection based on the Banff criteria (24), as determined by transplant pathologists on allograft kidney biopsy specimens. Renal biopsies included both protocol and indication biopsies, with indication biopsies more common in clinical practice. The clinically diagnosed acute rejection was defined only in cases of acute rejection (25). It was based on a >20% increase in serum creatinine within 72 hours post-transplantation in recipients in whom renal allograft biopsy could not be performed due to technical or clinical constraints. After exclusion of alternative causes and in the presence of a high clinical suspicion for rejection, acute rejection was clinically diagnosed if serum creatinine levels demonstrated a marked decline in response to methylprednisolone and/or anti-thymocyte globulin (ATG). In recipients with moderate-to-strong donor-specific antibody (DSA) positivity accompanied by a concomitant rise in serum creatinine, acute rejection was diagnosed if both serum creatinine and DSA levels decreased following ABMR-directed therapy such as plasma exchange, high-dose intravenous immunoglobulin (IVIG), and rituximab.

The Activity Index (AI) and Chronicity Index (CI) were defined and calculated according to the Banff lesion scoring system (22), as proposed by the Banff Activity and Chronicity Indices Working Group. AI was defined as i + t + v + g + ptc + C4d, where each lesion (i, t, v, g, ptc) was graded on a 0–3 scale and C4d was dichotomized as 0 (absent) or 2 (present), yielding a total AI score ranging from 0 to 17. CI was defined as ci + ct + cv + 2×cg, with each lesion graded on a 0–3 scale and cg weighted by a factor of 2, resulting in a total CI score ranging from 0 to 15. AI was initially categorized as low (0–4), moderate (5–9), and high (≥10), and CI as low (<4) and high (≥4). For combined analysis, AI was dichotomized into low/moderate (<10) and high (≥10) activity, and CI into low (<4) and high (≥4) chronicity. Recipients were subsequently stratified into four composite categories: Low AI/Low CI, High AI/Low CI, Low AI/High CI, and High AI/High CI.

### Treatment protocol for rejection episodes

For acute TCMR, the standard treatment involves intravenous methylprednisolone at a dose of 10 mg/kg/day for 3 days. The dosage and duration of ATG treatment are determined based on the clinical manifestations, laboratory findings, and the histologic grade of TCMR. Typically, ATG is administered at 1.5 mg/kg/day for 3–5 days, with the dosage adjusted according to recipient weight and clinical response. In cases of refractory rejection, the duration of ATG treatment may be extended to 5–10 days. For chronic active TCMR, treatment is based on the serum creatinine levels and the degree of chronic pathological changes. Usually, intravenous methylprednisolone is given at a dose of 5–10 mg/kg/day for 3 days. For ABMR, the treatment schedule is individualized according to clinical presentation, DSA levels, the presence of concomitant TCMR, and the extent of chronic damage. In cases of active ABMR, plasma exchange and high-dose IVIG (2 g/kg) are initiated, followed by a combination of methylprednisolone (10 mg/kg/day for 3 days) and/or rituximab, bortezomib, and other immunosuppressive treatments. For chronic active ABMR, treatment typically includes high-dose IVIG (1–2 g/kg) or a combination of methylprednisolone (5–10 mg/kg/day for 3 days) and/or rituximab, along with additional therapies aimed at managing ABMR, such as bortezomib or other agents, depending on the clinical situation. Subsequently, appropriate adjustments in the dosage and concentration of calcineurin inhibitors (tacrolimus, cyclosporine) and/or mycophenolate mofetil and oral glucocorticoids are made based on dynamic monitoring.

### Statistical analysis

Categorical variables were characterized by percentages and compared with chi-square tests or Fisher’s exact test. Continuous variables were tested for normality by the Shapiro–Wilk test, presented as mean ± SD or median (IQR), and compared using the Student’s t test or Mann–Whitney U test, as appropriate. Univariate logistic regression was used to identify potential risk factors for rejection, and variables with p < 0.1 were included in the multivariate logistic regression model. Odds ratios (ORs) and 95% confidence intervals were reported. The cumulative incidence of rejection and death-censored graft survival were analyzed by Kaplan–Meier methods with group comparisons using the log-rank test. Differences in Activity Index (AI) and Chronicity Index (CI) among rejection phenotypes were assessed by the Kruskal–Wallis test with Bonferroni-adjusted pairwise comparisons. For factors associated with high CI (≥4), univariate and multivariate logistic regression analyses were performed. All analyses were conducted using R software (version 4.3.1; R Foundation for Statistical Computing, Vienna, Austria), and p < 0.05 was considered statistically significant.

## Results

### Donor and recipient characteristics

**Figure 1:**
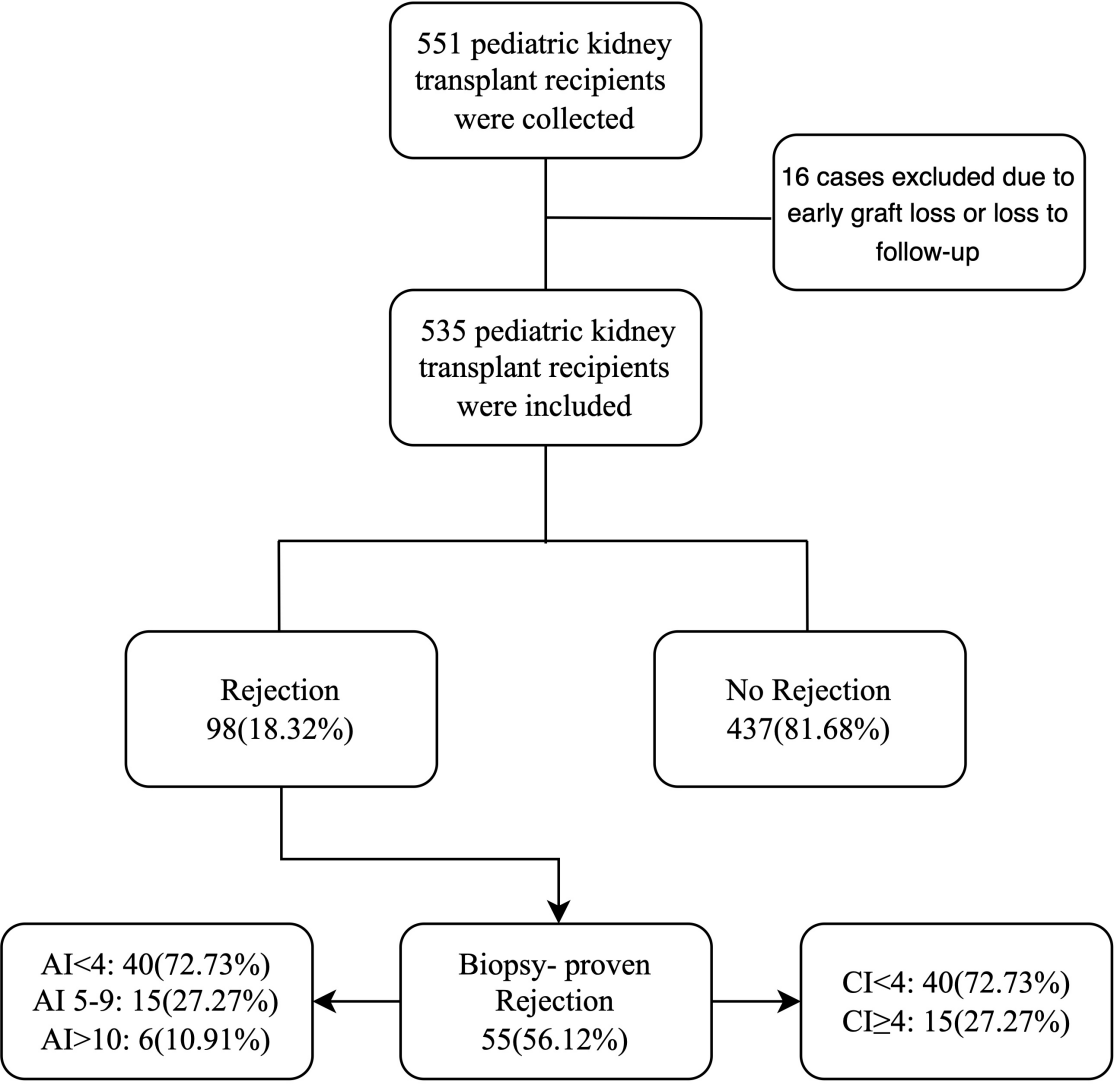
Flowchart illustrating the selection of pediatric kidney transplant recipients and classification by rejection status and AI/CI scores. AI, activity index; CI, chronicity index.

The study flowchart is presented in **Figure 1**. A total of 535 kidney transplant recipients were included in the analysis, comprising 98 recipients who experienced rejection and 437 without rejection. Donor and recipient characteristics are summarized in **Table 1**. Compared with the non-rejection group, recipients with rejection more frequently received grafts from younger donors (3.0 vs. 5.0 years, p = 0.047) and donors with lower body weight (14.75 vs. 18.00 kg, p = 0.023). In addition, the distribution of donor types differed significantly between the rejection and non-rejection groups (p = 0.041). Significant differences in HLA-B and HLA-DR mismatch were also observed between the two groups (p = 0.038 and p = 0.008, respectively). Moreover, a higher proportion of recipients with rejection had a history of previous transplantation compared with those without rejection (7.1% vs. 2.5%, p = 0.031). No other significant differences were found in donor or recipient characteristics between the two groups.

**Table 1:** Baseline characteristics of donors and recipients.

Characteristics	level	Rejection (n=98)	Non-rejection (n=437)	P value
Donor age, y, median [IQR]		3.00 [1.00, 11.00]	5.00 [2.00, 12.00]	0.047
Donor weight, kg, median [IQR]		14.75 [10.00, 31.50]	18.00 [12.00, 40.00]	0.023
Donor sex, n (%)	female	37 (37.8)	171 (39.1)	0.820
Donor HLA-A mismatch, n (%)	Fully mismatch	15 (15.3)	95 (21.7)	0.200
	Partial match	40 (40.8)	187 (42.8)	
	Fully match	43 (43.9)	155 (35.5)	
Donor HLA-B mismatch, n (%)	Fully mismatch	4 (4.1)	42 (9.6)	0.038
	Partial match	21 (21.4)	126 (28.8)	
	Fully match	73 (74.5)	269 (61.6)	
Donor HLA-DR mismatch, n (%)	Fully mismatch	2 (2.0)	48 (11.0)	0.008
	Partial match	32 (32.7)	161 (36.8)	
	Fully match	64 (65.3)	228 (52.2)	
Donor type	LD, n (%)	2 (2.0)	4 (0.9)	0.041
	DBD, n (%)	68 (69.4)	353 (80.8)	
	DCD, n (%)	28 (28.6)	80 (18.3)	
WIT, min, median [IQR]		1.00 [0.00, 2.00]	1.00 [0.00, 1.00]	0.094
CIT, hour, median [IQR]		8.00 [6.00, 10.00]	8.00 [5.50, 10.00]	0.538
Recipient age, median [IQR]		11.63 [8.65, 13.96]	11.71 [8.02, 15.10]	0.601
Recipient weight, kg, median [IQR]		29.25 [20.12, 36.75]	30.00 [19.00, 41.60]	0.334
Recipient sex, n (%)	female	50 (51.0)	186 (42.6)	0.144
Recipient primary disease, n (%)	CAKUT	16 (16.3)	57 (13.0)	0.381
	Cystic kidney disease	4 (4.1)	17 (3.9)	
	Glomerular disease	49 (50.0)	206 (47.1)	
	Hereditary / Tubulointerstitial disease	14 (14.3)	52 (11.9)	
	Metabolic disorder	0 (0.0)	11 (2.5)	
	Syndrome-related renal disease	6 (6.1)	54 (12.4)	
	unknown	9 (9.2)	40 (9.2)	
Dialysis type, n (%)	NO	29 (29.6)	128 (29.3)	0.367
	PD	29 (29.6)	159 (36.4)	
	HD	40 (40.8)	150 (34.3)	
Immune induction therapy, n (%)	ATG	62 (63.3)	244 (55.8)	0.214
	IL-2RA	36 (36.7)	193 (44.2)	
Maintenance immunosuppression, n (%)	Tacrolimus	92 (93.9)	396 (90.6)	0.429
	Cyclosporine	4 (4.1)	29 (6.6)	0.486
	mTOR Inhibitors	2 (2.0)	14 (3.2)	0.748
	MMF(vs EC-MPS)	49 (50.0)	189 (43.2)	0.261
	Steroid maintenance	64 (65.3)	265 (60.6)	0.423
DGF, n (%)		10 (10.2)	37 (8.5)	0.557
Recipient previous transplantation, n (%)		7 (7.1)	11 (2.5)	0.031
Combined organ transplantation, n (%)		0 (0.0)	11 (2.5)	0.229

LD, Living donor; DCD, Donation after circulatory death; WIT, Warm ischemia time; CIT, Cold ischemia time; CAKUT, Congenital anomalies of the kidney and urinary tract; PD, Peritoneal dialysis; HD, Hemodialysis; ATG, Anti-thymocyte globulin; IL-2RA, Interleukin-2 receptor antagonist; mTOR, Mammalian target of rapamycin; MMF, Mycophenolate mofetil; EC-MPS, Enteric-coated mycophenolic sodium; DGF, Delayed graft function.

### Incidence of rejection in pediatric kidney transplant recipients

Among 535 pediatric kidney transplant recipients, a total of 98 patients (18.3%) experienced 126 rejection episodes. Of these, 74 episodes (58.7%) were biopsy-proven rejections, whereas 52 episodes (41.3%) were clinically acute rejections. Among the biopsy-proven rejection, histopathologic subtypes included TCMR in 34 cases (46.0%; including 6 borderline TCMR), ABMR in 18 cases (24.3%), and mixed cellular and antibody-mediated rejection (mixed rejection) in 22 cases (29.7%). The first rejection occurred 7 days to 9.25 years after transplantation (median, 238 days). When limited to biopsy-proven episodes, rejection was observed later, occurring 16 days to 9.25 years post-transplant (median, 300 days). The cumulative incidence of rejection increased progressively over time, reaching 3.4% (95% CI, 1.8–4.9%) at 1 month, 5.4% (95% CI, 3.5–7.3%) at 3 months, 7.9% (95% CI, 5.6–10.1%) at 6 months, 10.8% (95% CI, 8.1–13.4%) at 1 year, 14.2% (95% CI, 11.1–17.1%) at 2 years, 16.3% (95% CI, 12.9–19.5%) at 3 years, and 19.2% (95% CI, 15.3–22.9%) at 5 years.

### Risk factor analysis for pediatric kidney transplant rejection

To identify factors associated with rejection in pediatric kidney transplant recipients, univariate and multivariate logistic regression analyses were performed (**Table 2**). In the univariate analysis, lower donor weight (OR = 0.99, 95% CI 0.97–1.00, p = 0.038), greater HLA-B mismatch (OR = 1.66, 95% CI 1.14–2.51, p = 0.012), HLA-DR mismatch (OR = 1.77, 95% CI 1.23–2.63, p = 0.003), longer warm ischemia time (WIT) (OR = 1.04, 95% CI 1.00–1.09, p = 0.047), history of previous transplantation (OR = 2.98, 95% CI 1.07–7.77, p = 0.028), DCD (OR = 1.79, 95% CI 1.07–2.92, p = 0.023), presence of preformed donor-specific antibodies (pfDSA) (OR = 2.51, 95% CI 0.92–6.30, p = 0.057), and recipient age between 9 and 15 years (OR = 1.88, 95% CI 1.21–2.94, p = 0.005) were associated with an increased risk of rejection.

**Table 2:** Risk factors for the occurrence of rejection in pediatric kidney transplant recipients.

Risk factors	Univariable analysis		Multivariable analysis	
	OR (CI)	P value	OR (CI)	P value
Donor age	0.98 (0.95–1.01)	0.262	–	–
Donor weight	0.99 (0.97–1.00)	0.038	0.99 (0.97–1.00)	0.072
Donor HLA-A mismatch	1.32 (0.98–1.80)	0.074	1.17 (0.85–1.61)	0.347
Donor HLA-B mismatch	1.66 (1.14–2.51)	0.012	1.50 (1.01–2.32)	0.055
Donor HLA-DR mismatch	1.77 (1.23–2.63)	0.003	1.60 (1.09–2.43)	0.021
WIT	1.04 (1.00–1.09)	0.047	1.03 (0.97–1.10)	0.291
CIT	1.01 (0.96–1.08)	0.631	–	–
Recipient age	0.99 (0.94–1.04)	0.588	–	–
Recipient weight	0.99 (0.97–1.01)	0.255	–	–
Recipient sex	0.71 (0.46–1.10)	0.129	–	–
Recipient previous transplantation	2.98 (1.07–7.77)	0.028	3.05 (1.03–8.53)	0.036
LD	2.26 (0.31–11.73)	0.352	–	–
DCD donor (vs. non-DCD)	1.79 (1.07–2.92)	0.023	1.27 (0.62–2.52)	0.505
pfPRA	0.94 (0.51–1.66)	0.842	–	–
pfDSA	2.51 (0.92–6.30)	0.057	3.11 (1.08–8.31)	0.027
Immune induction therapy IL-2RA (vs. ATG)	0.73 (0.46–1.15)	0.18	–	–
DGF	1.23 (0.56–2.48)	0.584	–	–
9–15 years old	1.88 (1.21–2.94)	0.005	2.05 (1.30–3.29)	0.002
Tacrolimus	1.59 (0.70–4.26)	0.307	–	–
Cyclosporine	0.60 (0.17–1.57)	0.347	–	–
MMF(vs EC-MPS)	0.76 (0.49–1.18)	0.225	–	–
Steroid maintenance	1.22 (0.78–1.95)	0.391	–	–
Tacrolimus trough <5 ng/mL (first 6 months post-transplant)	1.97 (0.68–5.05)	0.176	–	–

pfPRA, pre-formed Panel Reactive Antibodies; pfDSA, pre-formed Donor-Specific Antibodies.

In the multivariate model, HLA-DR mismatch (OR = 1.60, 95% CI 1.09–2.43, p = 0.021), history of previous transplantation (OR = 3.05, 95% CI 1.03–8.53, p = 0.036), presence of pfDSA (OR = 3.11, 95% CI 1.08–8.31, p = 0.027), and recipient age 9–15 years at transplantation (OR = 2.05, 95% CI 1.30–3.29, p = 0.002) remained significant independent predictors, while donor weight, WIT, and HLA-B mismatch were not significant in the adjusted model.

### Clinical outcomes in pediatric kidney transplant recipients with different rejection phenotypes

**Figure 2:**
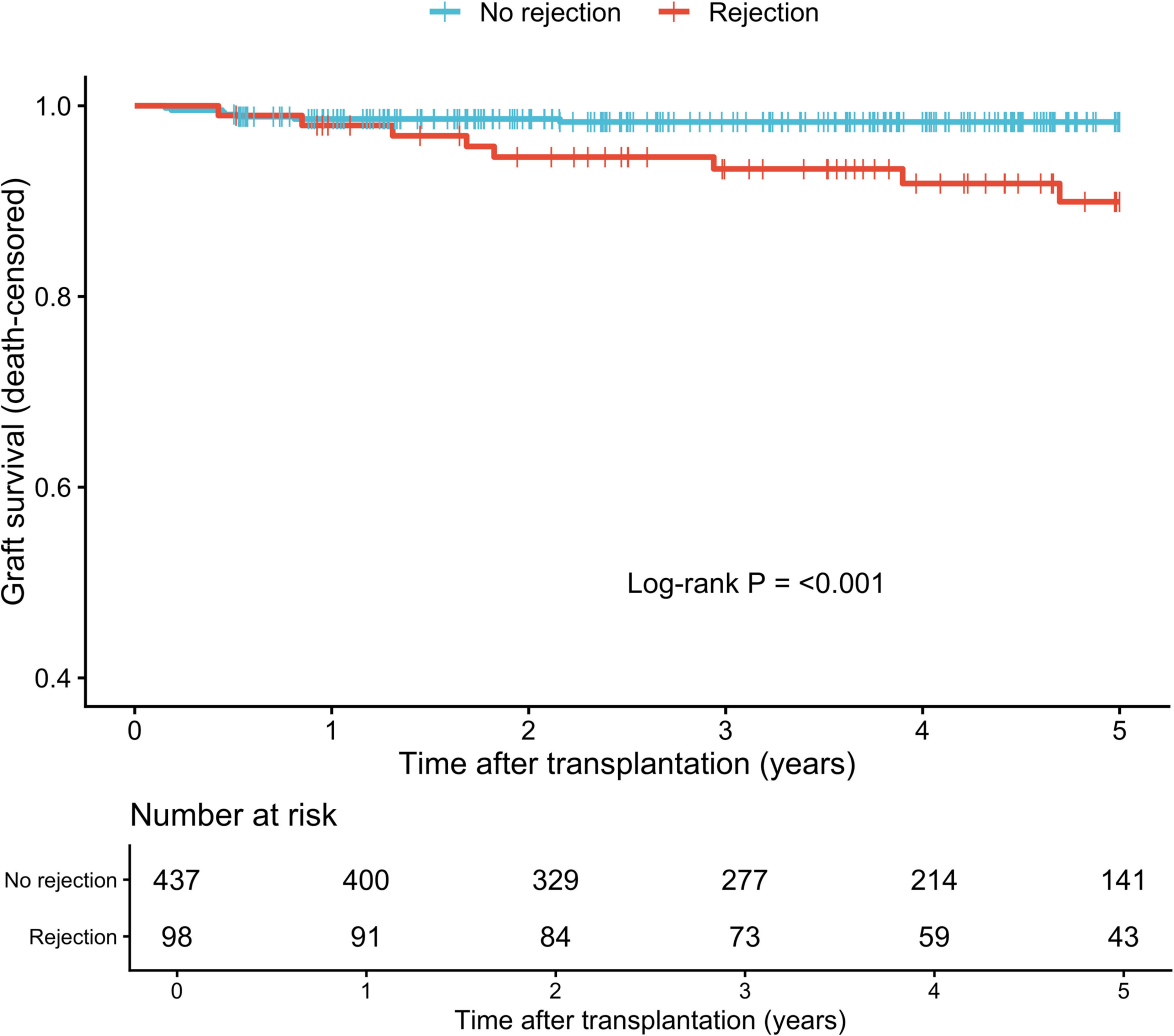
Kaplan-Meier plots of pediatric kidney transplantation grouped by rejection. Kaplan-Meier survival analysis between the non-rejection and rejection groups. Light blue: No rejection group; Red: Rejection group.

Kaplan–Meier survival analysis demonstrated that recipients with rejection had significantly lower death-censored graft survival compared with those without rejection (log-rank p < 0.001; **Figure 2**). At 5 years post-transplantation, graft survival was 89.9% (95% CI, 83.4–97.0%) in the rejection group and 98.3% (95% CI, 97.1–99.6%) in the non-rejection group. However, when further stratified by histological rejection phenotype, no statistically significant differences in graft survival were observed among patients with ABMR, TCMR, and mixed rejection (p = 0.29; **Supplementary Figure 1A**). Similarly, no significant survival differences were found across TCMR subcategories (p = 0.58; **Supplementary Figure 1B**).

**Figure 3:**
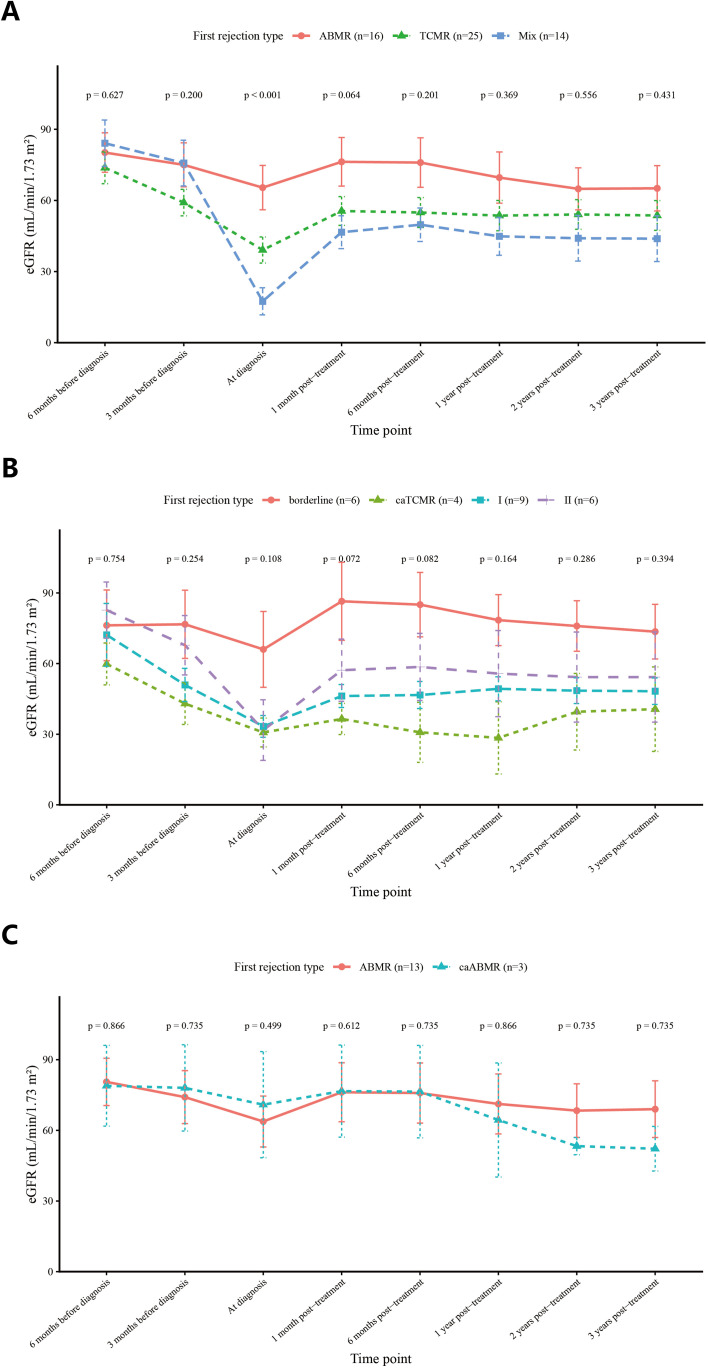
eGFR-time point curves of ABMR and TCMR groups. **(A)** eGFR levels at different time points in ABMR, TCMR, and Mixed rejection groups. Red curve: ABMR group; Green curve: TCMR group; Blue curve: Mixed rejection group. **(B)** eGFR levels at different time points in TCMR groups. Red curve: borderline group; Green curve: caTCMR group; Blue curve: TCMR grade I group; Purple curve: TCMR grade II group. **(C)** eGFR levels at different time points in ABMR groups. Red curve: active ABMR (aABMR) group; Cyan curve: chronic active ABMR (caABMR) group.

Based on estimated glomerular filtration rate (eGFR), we compared allograft function at multiple time points across rejection phenotypes (**Figure 3A**). A statistically significant difference in eGFR was observed at diagnosis (ABMR: 65.4 vs. TCMR: 39.1 vs. Mixed rejection: 17.5 mL/min/1.73 m²; p < 0.001). In contrast, no significant inter-group differences were found at any other time point: 6 months prior to diagnosis (ABMR: 80.2 vs. TCMR: 73.8 vs. Mixed rejection: 84.1 mL/min/1.73 m²; p = 0.627), 3 months prior to diagnosis (ABMR: 75.0 vs. TCMR: 59.1 vs. Mixed rejection: 75.8 mL/min/1.73 m²; p = 0.200), 1 month post-diagnosis (ABMR: 76.3 vs. TCMR: 55.5 vs. Mixed rejection: 46.6 mL/min/1.73 m²; p = 0.064), 6 months post-diagnosis (ABMR: 76.0 vs. TCMR: 54.9 vs. Mixed rejection: 49.8 mL/min/1.73 m²; p = 0.201), 1 year post-diagnosis (ABMR: 69.6 vs. TCMR: 53.6 vs. Mixed rejection: 44.9 mL/min/1.73 m²; p = 0.369), 2 years post-diagnosis (ABMR: 64.9 vs. TCMR: 54.1 vs. Mixed rejection: 44.1 mL/min/1.73 m²; p = 0.556), and 3 years post-diagnosis (ABMR: 65.1 vs. TCMR: 53.7 vs. Mixed rejection: 43.9 mL/min/1.73 m²; p = 0.431). Furthermore, among recipients with ABMR or mixed rejection, stratification according to *de novo* donor-specific antibody (dnDSA) status revealed no significant differences in eGFR between dnDSA-positive and dnDSA-negative subgroups (**Supplementary Figure 2**).

### Prognostic value of activity and chronicity index in pediatric kidney transplantation

**Figure 4:**
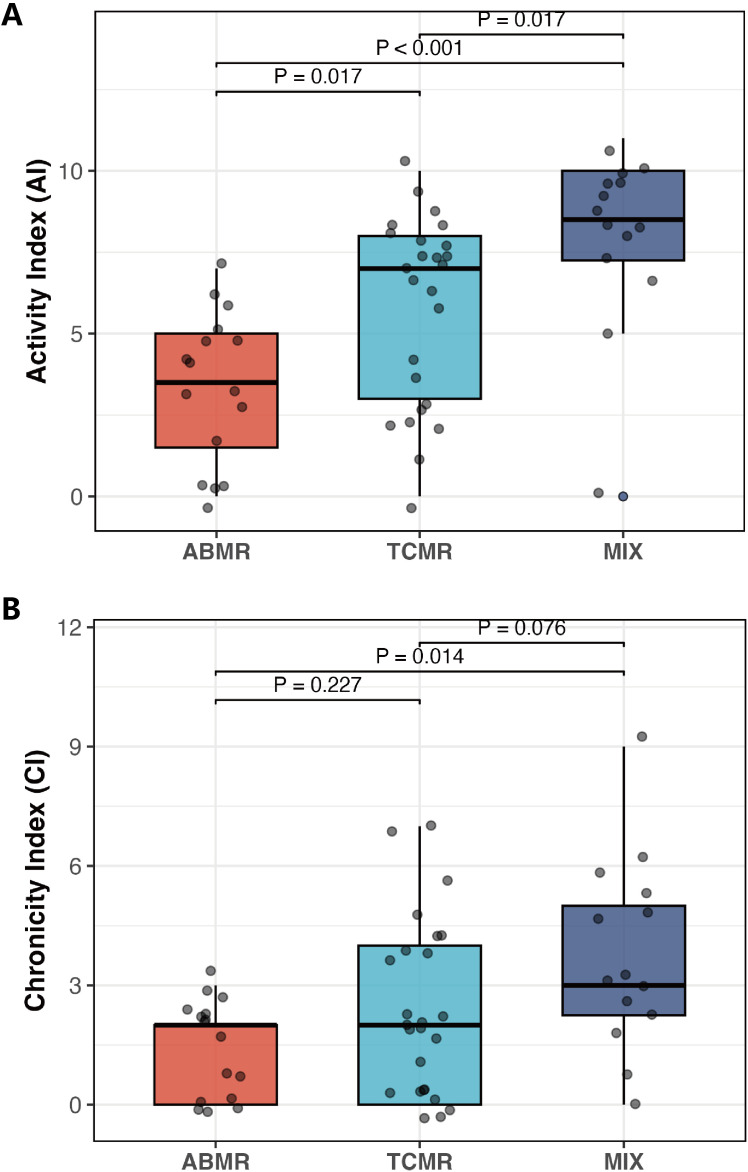
Activity index (AI) and Chronicity index (CI) in ABMR/TCMR or Mixed rejection group. **(A)** AI score in three group; **(B)** CI score in three group.

To assess the distribution of activity and chronicity scores across different rejection phenotypes, we compared AI and CI among ABMR, TCMR, and mixed rejection groups. As shown in **Figure 4A**, the median AI scores were significantly different among the three groups (Kruskal–Wallis p < 0.001). Recipients with mixed rejection had the highest median AI [8.5 (IQR 7.2–10.0)], followed by TCMR [7.0 (IQR 3.0–8.0)] and ABMR [3.5 (IQR 1.5–5.0)]. Pairwise comparisons revealed significantly higher AI in TCMR vs ABMR (7.0 vs 3.5, p = 0.017), Mixed rejection vs ABMR (8.5 vs 3.5, p < 0.001), and Mixed rejection vs TCMR (8.5 vs 7.0, p = 0.017). For CI (**Figure 4B**), the overall difference among the three groups was also statistically significant (Kruskal–Wallis p = 0.017). The mixed rejection group exhibited the highest CI [3.0 (IQR 2.2–5.0)], compared with TCMR [2.0 (IQR 0.0–4.0)] and ABMR [2.0 (IQR 0.0–2.0)]. Pairwise analysis showed that CI was significantly higher in Mixed rejection vs ABMR (3.0 vs 2.0, p = 0.014), whereas the differences between TCMR vs ABMR (2.0 vs 2.0, p = 0.227) and Mixed rejection vs TCMR (3.0 vs 2.0, p = 0.076).

**Figure 5:**
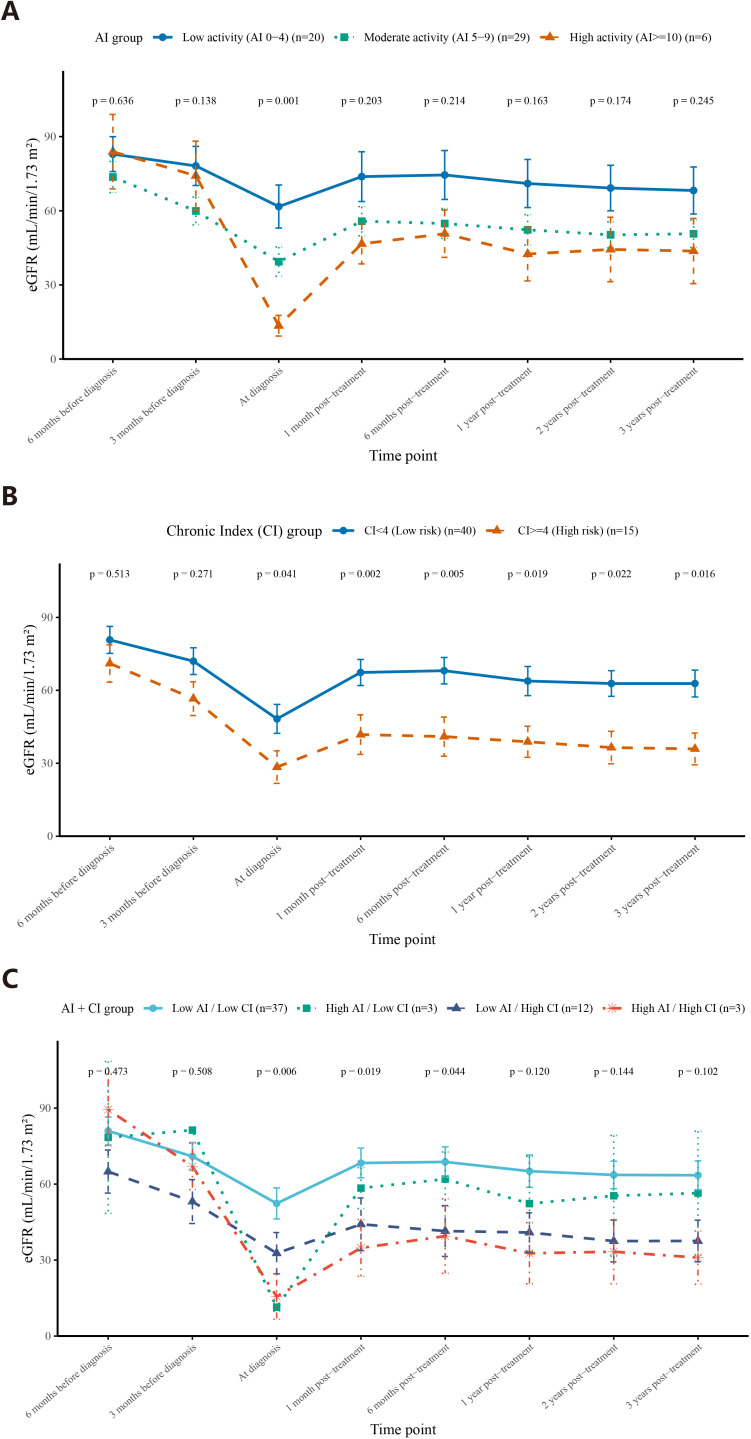
eGFR-time point curves of different group defined with AI score and/or CI score. **(A)** eGFR levels at different time points in subgroups defined by AI score. Blue curve: Low activity (AI 0-4); Green curve: Moderate activity (AI 5-9); Orange curve: High activity (AI≥10). **(B)** eGFR levels at different time points in subgroups defined by CI score. Blue curve: Low risk group (CI<4); Orange curve: High risk group (CI≥4). **(C)** eGFR levels at different time points in subgroups defined by AI score and CI score. Light blue curve: Low AI/ Low CI group (AI < 10, CI < 4); Green curve: High AI/ Low CI group (AI ≥ 10, CI < 4); Dark blue curve: Low AI/ High CI group (AI < 10, CI ≥ 4); Orange curve: High AI/ High CI group (AI ≥ 10, CI ≥ 4).

To evaluate the prognostic value of histological injury burden, eGFR trends were compared using both traditional Banff rejection classification and semiquantitative AI and CI scores. In the analysis based on Banff classification (**Figures 3B, C**), no significant differences in eGFR were observed between rejection phenotypes, including ABMR vs caABMR and TCMR vs caTCMR, at any time point before or after rejection episodes. When recipients were stratified by CI scores, a clear and sustained separation of eGFR trajectories was observed. eGFR remained consistently lower in recipients with high CI (≥4) than in those with low CI (<4) at nearly all time points (6 months before diagnosis: 71.1 vs. 80.7 mL/min/1.73 m², p = 0.513; 3 months before: 56.6 vs. 72.0, p = 0.271; at diagnosis: 28.4 vs. 48.3, p = 0.041; 1 month post-treatment: 41.8 vs. 67.3, p = 0.002; 1 year: 38.8 vs. 63.8, p = 0.019; 3 years: 35.9 vs. 62.8, p = 0.016) (**Figure 5B**), underscoring the long-term prognostic impact of chronic histological injury. In contrast, stratification by AI scores primarily reflected acute functional impairment. Baseline eGFR values were comparable among the low (AI 0–4), moderate (AI 5–9), and high (AI ≥10) activity groups (p = 0.913 and 0.348 at 6 and 3 months before diagnosis, respectively). However, eGFR at diagnosis progressively declined with increasing AI severity (low: 61.7 vs. moderate: 39.4 vs. high: 13.5 mL/min/1.73 m²; p = 0.001). This difference was most evident at diagnosis and during the early post-treatment phase but gradually diminished during follow-up (**Figure 5A**). When AI and CI were jointly analyzed (four-group model: Low AI/Low CI, High AI/Low CI, Low AI/High CI, and High AI/High CI), eGFR at diagnosis differed significantly among the four groups (52.4 vs. 43.8 vs. 34.8 vs. 15.6 mL/min/1.73 m²; p = 0.006) (**Figure 5C**). This difference remained significant at 1 month (p = 0.019) and 6 months (p = 0.044) after treatment but was not significant thereafter, highlighting the complementary roles of AI and CI in capturing both acute and chronic components of graft injury. We further examined the association between dnDSA and histological severity (**Supplementary Table 1**). No significant differences in dnDSA intensity were observed across AI, CI, or combined AI/CI categories. Recipients were stratified into four groups according to combined CI score and dnDSA status (CI<4/dnDSA−, CI<4/dnDSA+, CI≥4/dnDSA−, and CI≥4/dnDSA+). As shown in **Supplementary Figure 5**, eGFR trajectories were primarily separated by CI stratification. Recipients with CI≥4 consistently exhibited lower eGFR across follow-up, irrespective of dnDSA status, whereas dnDSA positivity within the same CI category did not result in sustained differences in graft function. Consistently, Kaplan–Meier analysis showed no significant difference in death-censored graft survival among the four subgroups (p = 0.72).

To explore factors associated with high CI scores at the first biopsy, recipients were classified into a low CI group (CI <4) and a high CI group (CI ≥4), and baseline characteristics were compared between the two groups (**Table 3**). Donor type differed significantly between the groups (P = 0.042), with a higher proportion of DCD donors in the high CI group. The remaining clinical variables were comparable between the two groups (**Table 3**).

**Table 3:** Baseline characteristics of pediatric kidney transplant recipients with rejection stratified by CI score.

Factors	Level	CI<4 (Low risk) (n=40)	CI≥4 (High risk) (n=15)	P value
Donor age, y, median [IQR]		3.00 [1.00, 12.50]	8.00 [3.00, 15.00]	0.139
Donor weight, kg, median [IQR]		16.00 [9.81, 45.00]	20.00 [11.00, 37.50]	0.432
Recipient age, y, median [IQR]		12.44 [9.19, 14.07]	11.62 [10.66, 14.06]	0.734
Recipient weight, kg, median [IQR]		31.00 [19.88, 40.25]	34.00 [27.75, 38.00]	0.508
Recipient sex, n (%)	Female	20 (50.0)	9 (60.0)	0.558
	Male	20 (50.0)	6 (40.0)	
Recipient primary disease, n (%)	CAKUT	8 (20.0)	1 (6.7)	0.248
	Cystic kidney disease	1 (2.5)	0 (0.0)	
	Glomerular disease	20 (50.0)	12 (80.0)	
	Hereditary / Tubulointerstitial disease	5 (12.5)	0 (0.0)	
	Syndrome-related renal disease	3 (7.5)	0 (0.0)	
	unknown	3 (7.5)	2 (13.3)	
Donor HLA-A mismatch, n (%)	Fully mismatch	5 (12.5)	0 (0.0)	0.089
	Partial match	17 (42.5)	11 (73.3)	
	Fully match	18 (45.0)	4 (26.7)	
Donor HLA-B mismatch, n (%)	Fully mismatch	2 (5.0)	1 (6.7)	0.273
	Partial match	4 (10.0)	4 (26.7)	
	Fully match	34 (85.0)	10 (66.7)	
Donor HLA-DR mismatch, n (%)	Fully mismatch	1 (2.5)	1 (6.7)	0.724
	Partial match	13 (32.5)	4 (26.7)	
	Fully match	26 (65.0)	10 (66.7)	
WIT, min, median [IQR]		1.00 [0.00, 3.00]	3.00 [1.00, 11.50]	0.058
CIT, hour, median [IQR]		8.00 [6.00, 10.00]	7.00 [5.50, 9.50]	0.228
Donor type	LD, n (%)	2 (5.0)	0 (0.0)	0.042
	DBD, n (%)	26 (65.0)	5 (33.3)	
	DCD, n (%)	12 (30.0)	10 (66.7)	
Dialysis type, n (%)	NO	11 (27.5)	6 (40.0)	0.666
	PD	9 (22.5)	3 (20.0)	
	HD	20 (50.0)	6 (40.0)	
Immune induction therapy, n (%)	ATG	27 (67.5)	9 (60.0)	0.752
	IL-2RA	13 (32.5)	6 (40.0)	
Maintenance immunosuppression, n (%)	Tacrolimus	37 (92.5)	14 (93.3)	1.00
	Cyclosporine	2 (5.0)	1(6.7)	1.00
	mTOR Inhibitors	1 (2.5)	0(0.0)	1.00
	MMF(vs EC-MPS)	23 (57.5)	10 (66.7)	0.758
	Steroid maintenance	25 (62.5)	12 (80.0)	0.335
DGF, n (%)		4 (10.0)	4 (26.7)	0.193
Recipient previous transplantation, n (%)		4 (10.0)	1 (6.7)	1.00
AI score, n (%)	Low (0–4)	16 (40.0)	2 (13.3)	0.169
	Moderate (5–9)	19 (47.5)	10 (66.7)	
	High (≥10)	5 (12.5)	3 (20.0)	

AI score, activity index score; CI score, chronic index score.

To explore the risk factors associated with high CI scores at the first biopsy, univariate and multivariate logistic regression analyses were performed for a CI score of ≥4. Univariable analysis identified both DCD and a time interval from transplantation to biopsy ≥3 years as significant predictors of high CI scores (OR = 4.67, 95% CI: 1.36–17.87, p = 0.017; OR = 4.57, 95% CI: 1.29–17.11, p = 0.02, respectively). In multivariable analysis, DCD (OR = 3.95, 95% CI: 1.08–15.86, p = 0.04) and biopsy performed ≥3 years after transplantation (OR = 3.80, 95% CI: 1.00–15.11, p = 0.05) remained independent risk factors for high CI scores at the first biopsy. Other clinical variables, including donor age, warm ischemia time, recipient age (9–15 years), and delayed graft function (DGF), were not significantly associated with high CI scores (**Table 4**).

**Table 4:** Risk factors for a chronicity index (CI) score ≥4 at the first biopsy.

Risk factors	Univariable analysis		Multivariable analysis	
	OR (CI)	P value	OR (CI)	P value
Time from transplantation to biopsy ≥3 years	4.57 (1.29–17.11)	0.02	3.80 (1.00,15.11)	0.05
Donor age	0.98 (0.95–1.01)	0.262	–	–
Donor HLA-A mismatch	0.86 (0.33–2.25)	0.759	–	–
Donor HLA-B mismatch	0.55 (0.19–1.55)	0.240	–	–
Donor HLA-DR mismatch	0.92 (0.33–2.85)	0.882	–	–
WIT	1.08 (0.98–1.19)	0.130	–	–
CIT	0.93 (0.79–1.08)	0.364	–	–
Recipient age	1.08 (0.92–1.29)	0.372	–	–
Recipient previous transplantation	0.64 (0.03–4.83)	0.704	–	–
LD	0.00 (NA–10^9)	0.993	–	–
DCD donor (vs. non-DCD)	4.67 (1.36–17.87)	0.017	3.95(1.08,15.86)	0.04
pfPRA	0.29 (0.01–1.78)	0.258	–	–
pfDSA	0.00 (NA–10^9)	0.992	–	–
9–15 years old	2.96 (0.79–14.47)	0.132	–	–
Immune induction therapy IL-2RA (vs. ATG)	1.38 (0.39–4.71)	0.603	–	–
DGF	3.27 (0.68–16.06)	0.132	–	–
dnDSA MFI level	1.33 (0.85–2.08)	0.211	–	–
Tacrolimus	1.14 (0.13–24.01)	0.916	–	–
Cyclosporine	1.36 (0.06–15.27)	0.809	–	–
mTOR Inhibitors	0.00 (NA–10^9)	0.992	–	–
MMF(vs EC-MPS)	0.44 (0.13–1.47)	0.189	–	–
Steroid maintenance	2.40 (0.64–11.79)	0.226	–	–

## Discussion

Acute rejection (AR) remains a major determinant of long-term outcomes in pediatric kidney transplantation. In our cohort, the 1-year incidence of AR was 10.8%, with TCMR as the predominant subtype. Kaplan–Meier analysis demonstrated that AR significantly impaired graft survival, and recurrent episodes further increased the risk of graft loss. We also identified several key risk factors for AR, including HLA-DR mismatch, preformed DSA, recipient previous transplantation, and adolescent age. In our pediatric kidney transplant cohort, only CI ≥ 4 showed a strong and persistent association with long-term graft outcomes, whereas AI was mainly related to short-term functional impairment at the time of rejection. These results emphasize that CI-based chronic injury assessment provides prognostic information for long-term graft survival and highlight the unique characteristics of rejection in pediatric recipients.

Acute rejection remains a major clinical challenge in pediatric kidney transplantation, and its incidence consistently exceeds that observed in adults. According to Organ Procurement and Transplantation Network (OPTN) data from 2021–2023, the 1-year post-transplant incidence of AR in pediatric recipients was 10%–11.4%, notably higher than the approximately 7% reported in adults (2, 26, 27). In our pediatric cohort, the 1-year cumulative incidence of AR was 10.8% (95% CI, 8.1%–13.4%), which aligns well with international reports. Among 74 biopsy-proven AR episodes in our study, TCMR accounted for nearly half of all biopsy-proven rejection episodes (45.95%). Similarly, a pediatric multicenter analysis found that T cell mediated rejection, including Banff grade I–III TCMR, caTCMR, and borderline rejection, accounted for 40.6% of all rejection diagnoses in the indication biopsy cohort (28). In addition, Kaplan–Meier survival analysis in our study demonstrated that AR was significantly associated with impaired graft survival. Recipients who experienced AR had markedly lower death-censored graft survival compared with those without. These observations are consistent with previous reports demonstrating that the occurrence of AR is strongly associated with graft loss (29–32). The strong negative association between repeated AR and long-term graft failure underscores the necessity of vigilant surveillance, timely intervention, and optimization of immunosuppressive therapy in pediatric recipients. In addition, recipients with ABMR exhibited lower Banff AI and CI scores compared with those with TCMR. This likely reflects the impact of routine DSA surveillance in our center, enabling earlier detection of subclinical or mild ABMR before irreversible damage occurs. In contrast, TCMR lacks sensitive noninvasive biomarkers (33–35), resulting in delayed clinical recognition and more advanced histopathologic injury at the time of diagnosis. These findings highlight the critical role of early immunologic surveillance in enabling timely intervention and mitigating irreversible graft injury.

Our study underscores that immunologic factors, particularly HLA-DR mismatch, preformed DSA, recipient previous transplantation, and adolescent recipient age, are the predominant determinants of rejection risk in pediatric kidney transplantation. HLA-DR mismatch and preformed DSA were strongly associated with biopsy-proven rejection, aligning with evidence from the CERTAIN pediatric registry showing that HLA-DR mismatch is an independent risk factor for ABMR, and preformed DSA also tends to increase the risk of rejection (36, 37). A history of previous transplantation, reflecting previous alloantigen exposure and immune priming, was also identified as an independent risk factor, likely due to heightened immunologic responsiveness in this population (36, 38, 39). Notably, recipient age between 9 and 15 years was associated with a higher risk, consistent with previous pediatric studies (40–42). This vulnerable period is often characterized by heightened immunologic responsiveness and suboptimal adherence during adolescence. Therefore, these findings indicate that immunologic burden and age-specific vulnerability jointly underlie the elevated rejection risk in pediatric recipients, highlighting the need for age-appropriate, risk-adapted management approaches.

In recent years, the Banff AI score and CI score have been introduced as quantitative adjuncts to the Banff classification, allowing for more standardized risk stratification and prognostic assessment. Traditional rejection categories have long been applied to classify kidney allograft rejection based on presumed pathobiologic mechanisms. However, these dichotomous categories and their subtypes such as active rejection, chronic active rejection, and chronic rejection are inherently heterogeneous and fail to fully capture the spectrum of rejection (22). Such variability complicates clinical decision-making and prognostic assessment (22, 43). To address these limitations, the Banff Foundation Working Group has emphasized AI and CI as objective measures that reflect acute and chronic allograft damage, respectively. In adult kidney transplantation, several studies have demonstrated the prognostic value of these indices (22, 44). Across multiple studies in adult kidney transplantation, CI has demonstrated consistent and robust prognostic value, whereas the predictive performance of AI remains controversial. For example, Matthias K. Haas et al. found that CI ≥ 4 was strongly associated with graft loss and remained significant in multivariable models, whereas AI showed no independent association, underscoring the stronger prognostic relevance of chronic injury over active inflammation (23). Similarly, another study focusing on late ABMR found that CI showed a borderline-significant association with graft loss (HR 1.97, 95% CI 0.97–3.99, p = 0.059), whereas AI was not an independent predictor of either eGFR slope or graft outcome, further emphasizing the limited prognostic value of activity indices compared with chronic injury scores (45). However, another study found that CI (per unit increase) was independently associated with graft loss (HR 1.24, 95% CI 1.09–1.42, p = 0.001), and AI ≥ 4 in ABMR was also an independent risk factor (HR 2.23, 95% CI 1.08–4.58, p = 0.029). Death-censored graft survival was significantly lower in patients with AI ≥ 4 or CI ≥ 4 (46). However, most evidence on AI and CI has been derived from adult populations. Whether these indices have comparable prognostic performance in pediatric kidney transplantation remains insufficiently studied. In our pediatric cohort, only CI demonstrated a sustained association with long-term renal functional decline, whereas AI was primarily related to short-term functional changes at the time of rejection. Although recipients with higher AI scores had significantly lower eGFR at the time of diagnosis, this difference was no longer significant during long-term follow-up. This suggests that while AI may reflect the short-term inflammatory burden and acute functional decline at the time of rejection, it is less predictive of subsequent renal trajectory once the acute process is controlled. In contrast, CI demonstrated a consistent and persistent association with renal function decline. Recipients with CI ≥ 4 had persistently lower eGFR from diagnosis through up to 3 years post-treatment. This durable difference underscores the role of CI as a marker of cumulative irreversible injury, aligning with observations in adult cohorts that have reported its strong association with long-term graft outcomes. In addition, no significant association was observed between dnDSA intensity and histological severity across AI, CI, or combined AI/CI categories. Recipients with CI ≥ 4 consistently exhibited lower eGFR during follow-up, irrespective of dnDSA status, whereas dnDSA positivity within the same CI category was not associated with sustained differences in graft function. Similarly, death-censored graft survival did not differ significantly among the four CI/dnDSA subgroups. Overall, dnDSA intensity was not independently associated with chronic injury burden or long-term graft outcomes in our cohort. Furthermore, high CI was associated with DCD donation in our study. The increased risk of high CI in DCD kidneys may reflect the long-term sequelae of early ischemia–reperfusion injury, which promotes microvascular loss, chronic hypoxia, and interstitial fibrosis (47, 48). Taken together, our findings indicate that CI provides long-term prognostic discrimination compared with AI, emphasizing the clinical importance of chronic injury burden for risk stratification, individualized follow-up, and prediction of long-term functional outcomes in pediatric kidney transplantation.

There are several limitations in this study. Firstly, it was a single-center retrospective analysis, which may affect the generalizability of the findings. Secondly, most biopsies in our cohort were indication biopsies rather than protocol biopsies, which might have introduced potential selection bias. Finally, the retrospective design precluded the systematic collection of objective medication adherence data and prevented full control over therapeutic heterogeneity across rejection phenotypes (e.g. TCMR vs. ABMR), which may have confounded graft outcomes despite standardized protocols.

In conclusion, our findings highlight the impact of acute rejection on long-term graft outcomes in pediatric kidney transplantation. AR remains more frequent in pediatric recipients than in adults and is associated with inferior graft survival. HLA-DR mismatch, preformed DSA, recipient previous transplantation, and adolescent recipient age were major risk factors for rejection. Furthermore, our findings further support the prognostic value of Banff activity and chronicity indices in pediatric kidney transplantation. While AI mainly reflects short-term inflammatory injury, CI ≥ 4 was more strongly and consistently associated with long-term functional decline, indicating stronger prognostic value than AI. These findings highlight the need for chronic injury–based risk assessment and early, tailored immunologic monitoring to better prevent and manage rejection in this population.

## Data Availability

The raw data supporting the conclusions of this article will be made available by the authors, without undue reservation.
