# Supplementary material for: Prognostic impact of rejection and chronicity index on long-term graft outcomes in pediatric kidney transplant recipients
Source: Front Immunol. 2026 Mar 20;17:1737410. doi: 10.3389/fimmu.2026.1737410 (PMC13046560; doi:10.3389/fimmu.2026.1737410)
Supplement: Supplementary file 1 [file DataSheet1.docx]

Supplementary Table S1. Association of dnDSA intensity with histological activity and chronicity indices

| Group | Total (n) | Negative | Weak | Moderate | Strong | P value |
| --- | --- | --- | --- | --- | --- | --- |
| Activity Index (AI) |  |  |  |  |  | 0.303 |
| Low (0–4) | 20 | 11 (55.0%) | 5 (25.0%) | 2 (10.0%) | 2 (10.0%) |  |
| Moderate (5–9) | 29 | 17 (58.6%) | 4 (13.8%) | 6 (20.7%) | 2 (6.9%) |  |
| High (≥10) | 6 | 2 (33.3%) | 0 (0.0%) | 2 (33.3%) | 2 (33.3%) |  |
| Chronicity Index (CI) |  |  |  |  |  | 0.078 |
| Low (<4) | 40 | 22 (55.0%) | 9 (22.5%) | 5 (12.5%) | 4 (10.0%) |  |
| High (≥4) | 15 | 8 (53.3%) | 0 (0.0%) | 5 (33.3%) | 2 (13.3%) |  |
| Combined AI/CI Categories |  |  |  |  |  | 0.108 |
| Low AI / Low CI | 37 | 21 (56.8%) | 9 (24.3%) | 4 (10.8%) | 3 (8.1%) |  |
| Low AI / High CI | 12 | 7 (58.3%) | 0 (0.0%) | 4 (33.3%) | 1 (8.3%) |  |
| High AI / Low CI | 3 | 1 (33.3%) | 0 (0.0%) | 1 (33.3%) | 1 (33.3%) |  |
| High AI / High CI | 3 | 1 (33.3%) | 0 (0.0%) | 1 (33.3%) | 1 (33.3%) |  |

AI, Activity Index; CI, Chronicity Index; dnDSA, de novo donor-specific antibody.

dnDSA intensity categories were defined based on peak MFI values: <1000 (negative), 1000–2999 (weak), 3000–4999 (moderate), and ≥5000 (strong).

Percentages were calculated within histological categories. Fisher’s exact test was used.


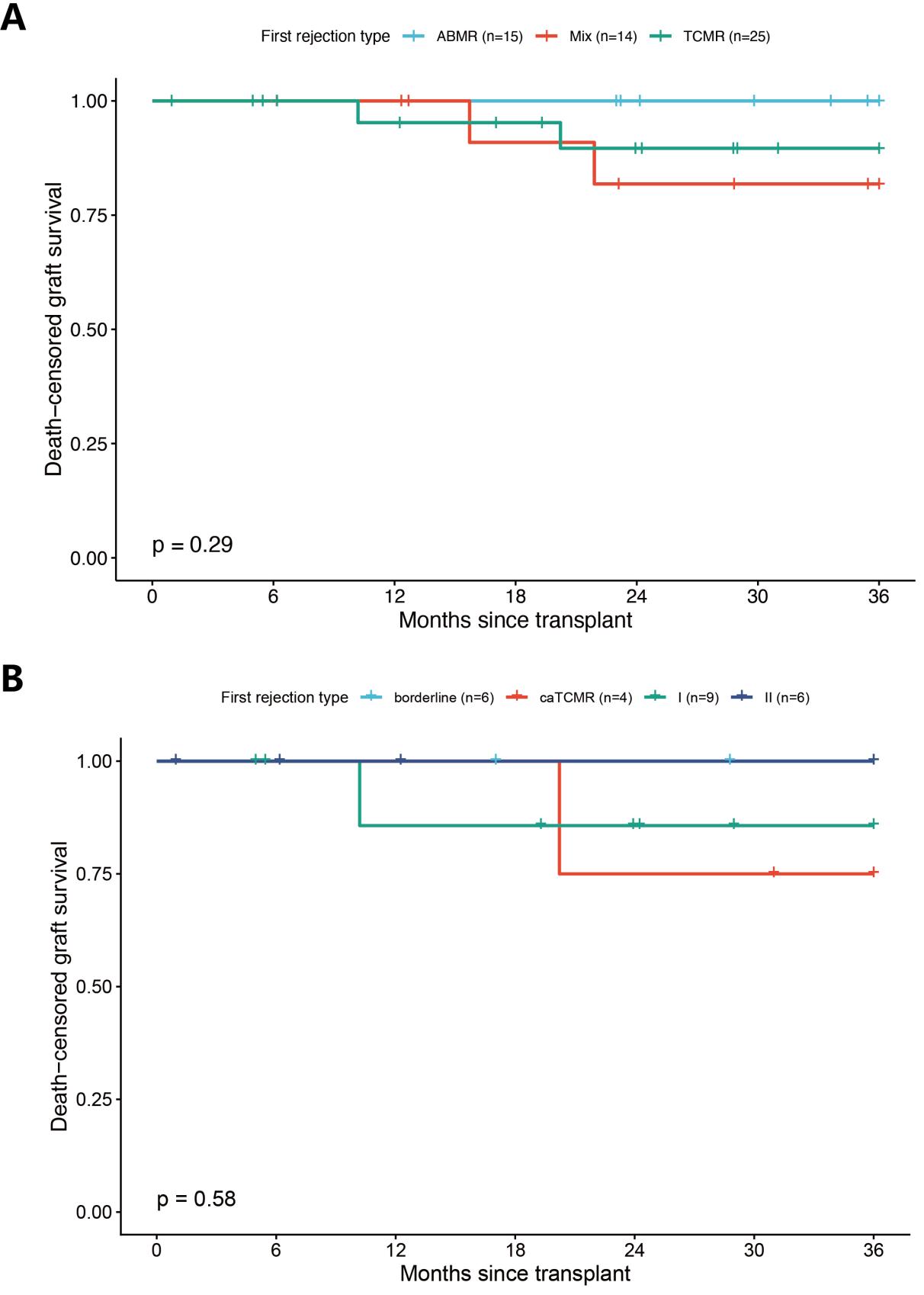


Figure.S1 Kaplan-Meier plots of different rejection group.

A Kaplan-Meier plot in ABMR\TCMR and Mix group. Cyan curve: ABMR group; Red curve: Mix group (ABMR + TCMR); Green curve: TCMR group. B, Kaplan-Meier plot within TCMR group. Cyan curve: borderline subgroup; Red curve: caTCMR subgroup; Green curve: TCMR stage I subgroup; Blue curve: TCMR stage II subgroup.


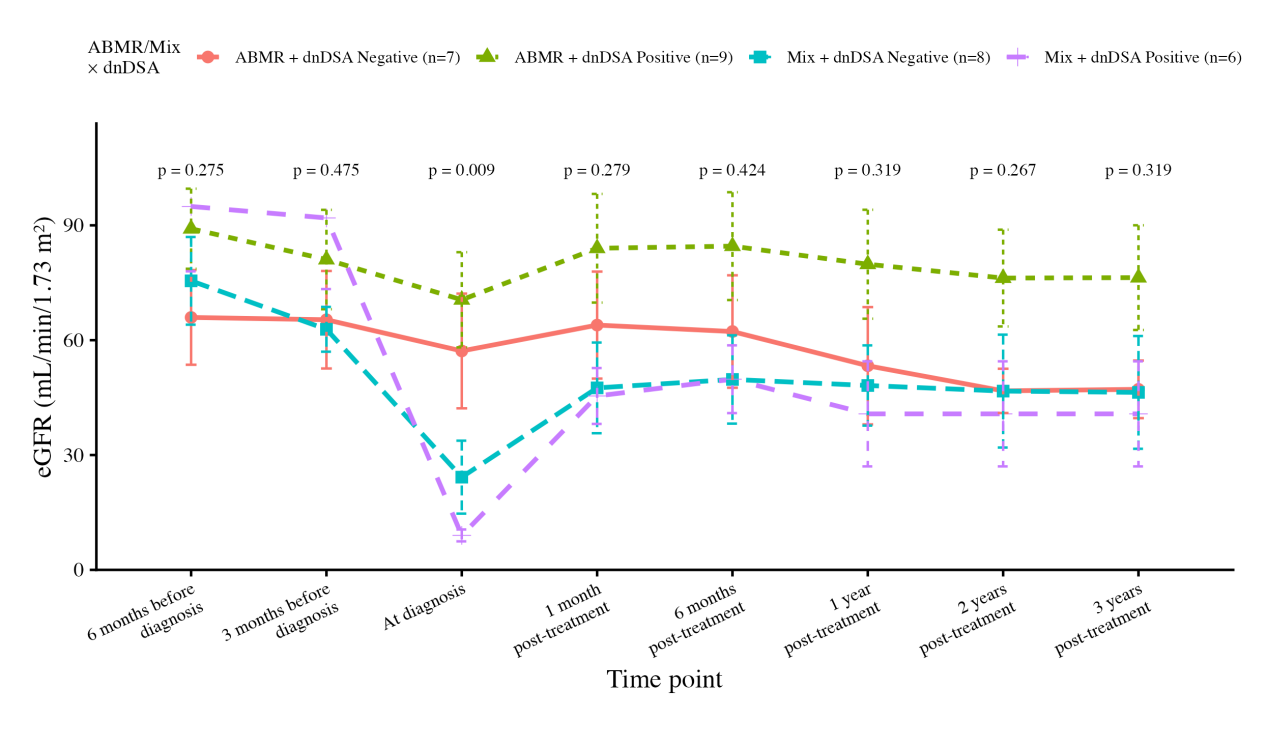


Figure.S2 eGFR trajectories over time in ABMR and mixed rejection groups stratified by dnDSA status. dnDSA, de novo donor-specific antibody. Red curve: ABMR&dnDSA- group; Green curve: ABMR&dnDSA+ group; Cyan curve: Mix&dnDSA- group; Purple curve: Mix&dnDSA+ group.


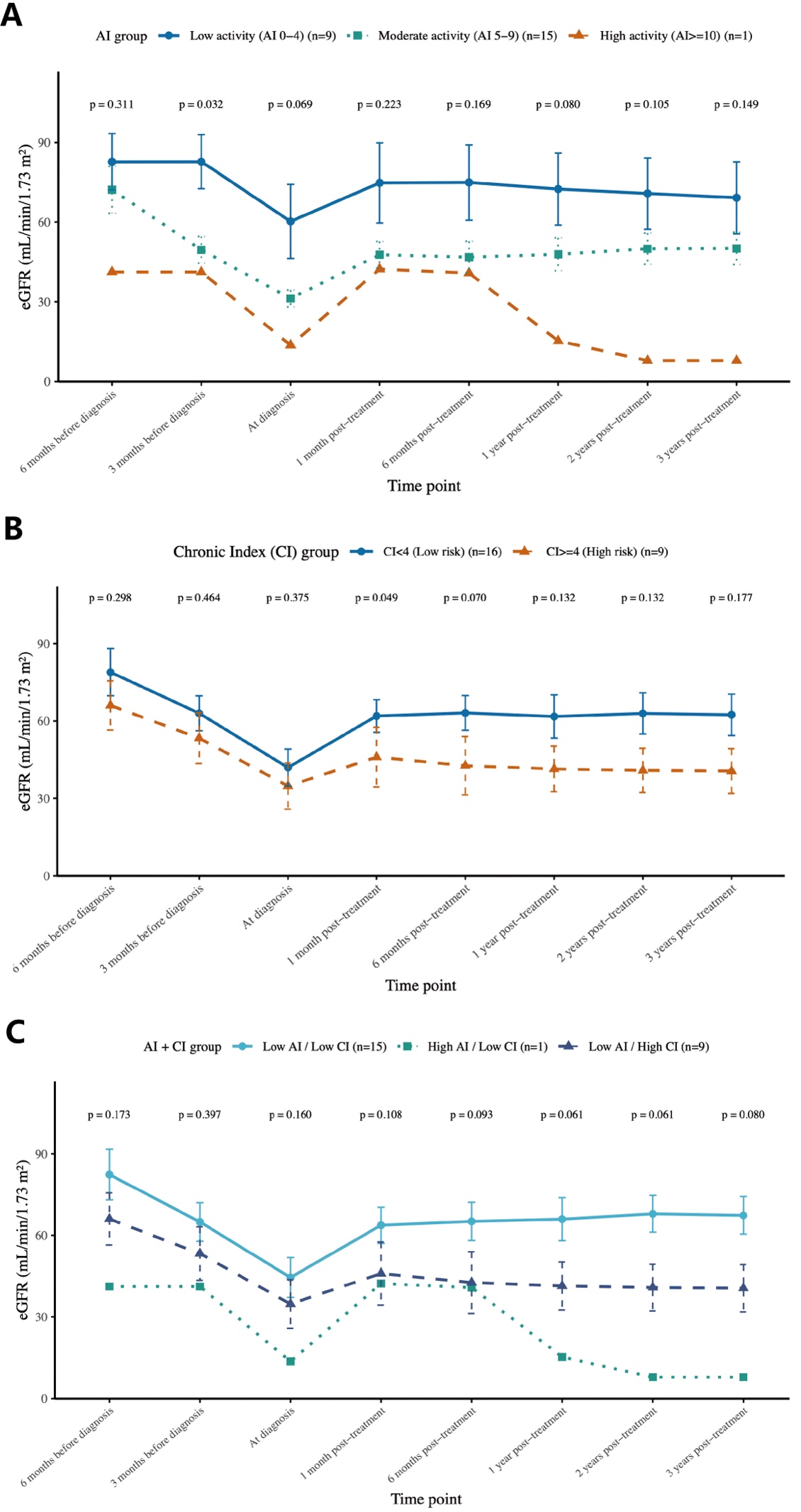


Figure.S3 eGFR-time point curves in TCMR subgroups defined with AI and CI score. A, eGFR levels at different time points in subgroups defined by AI score within TCMR. Blue curve: Low activity (AI 0-4); Green curve: Moderate activity (AI 5-9); Orange curve: High activity (AI≥10). B, eGFR levels at different time points in subgroups defined by CI score within TCMR. Blue curve: Low risk group (CI＜4); Orange curve: High risk group (CI≥4). C, eGFR levels at different time points in subgroups defined by AI score and CI score within TCMR. Light blue curve: Low AI/ Low CI group (AI < 10, CI < 4); Green curve: High AI/ Low CI group (AI ≥ 10, CI < 4); Dark blue curve: Low AI/ High CI group (AI < 10, CI ≥ 4).


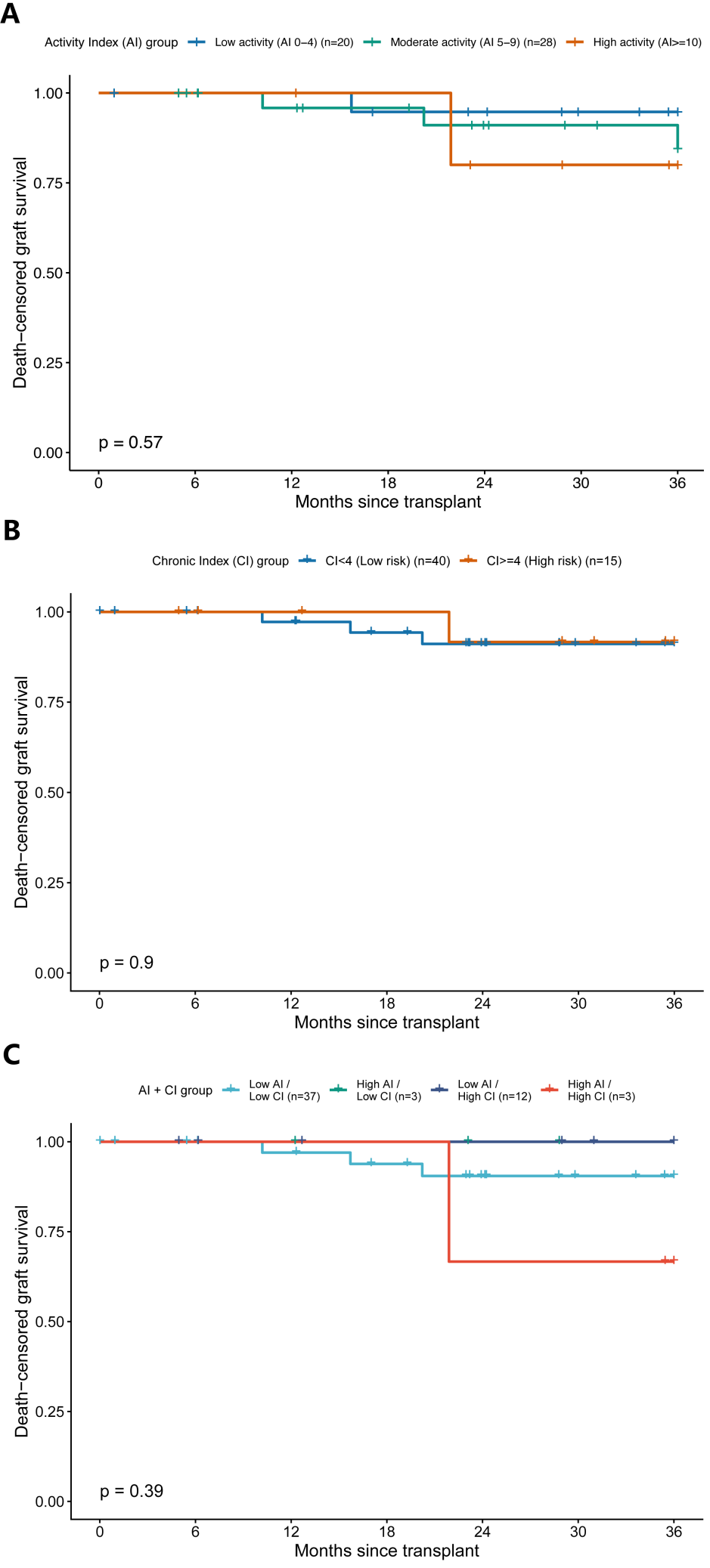


Figure.S4 Kaplan-Meier plots of different group defined with AI score or/and CI score.

A, Kaplan-Meier plot in subgroups defined by AI score. Blue curve: Low activity (AI 0-4); Green curve: Moderate activity (AI 5-9); Orange curve: High activity (AI≥10). B, Kaplan-Meier plot in subgroups defined by CI score. Blue curve: Low risk group (CI＜4); Orange curve: High risk group (CI≥4). C, Kaplan-Meier plot in subgroups defined by AI score and CI score. Light blue curve: Low AI/ Low CI group (AI < 10, CI < 4); Green curve: High AI/ Low CI group (AI ≥ 10, CI < 4); Dark blue curve: Low AI/ High CI group (AI < 10, CI ≥ 4); Orange curve: High AI/ High CI group (AI ≥ 10, CI ≥ 4).


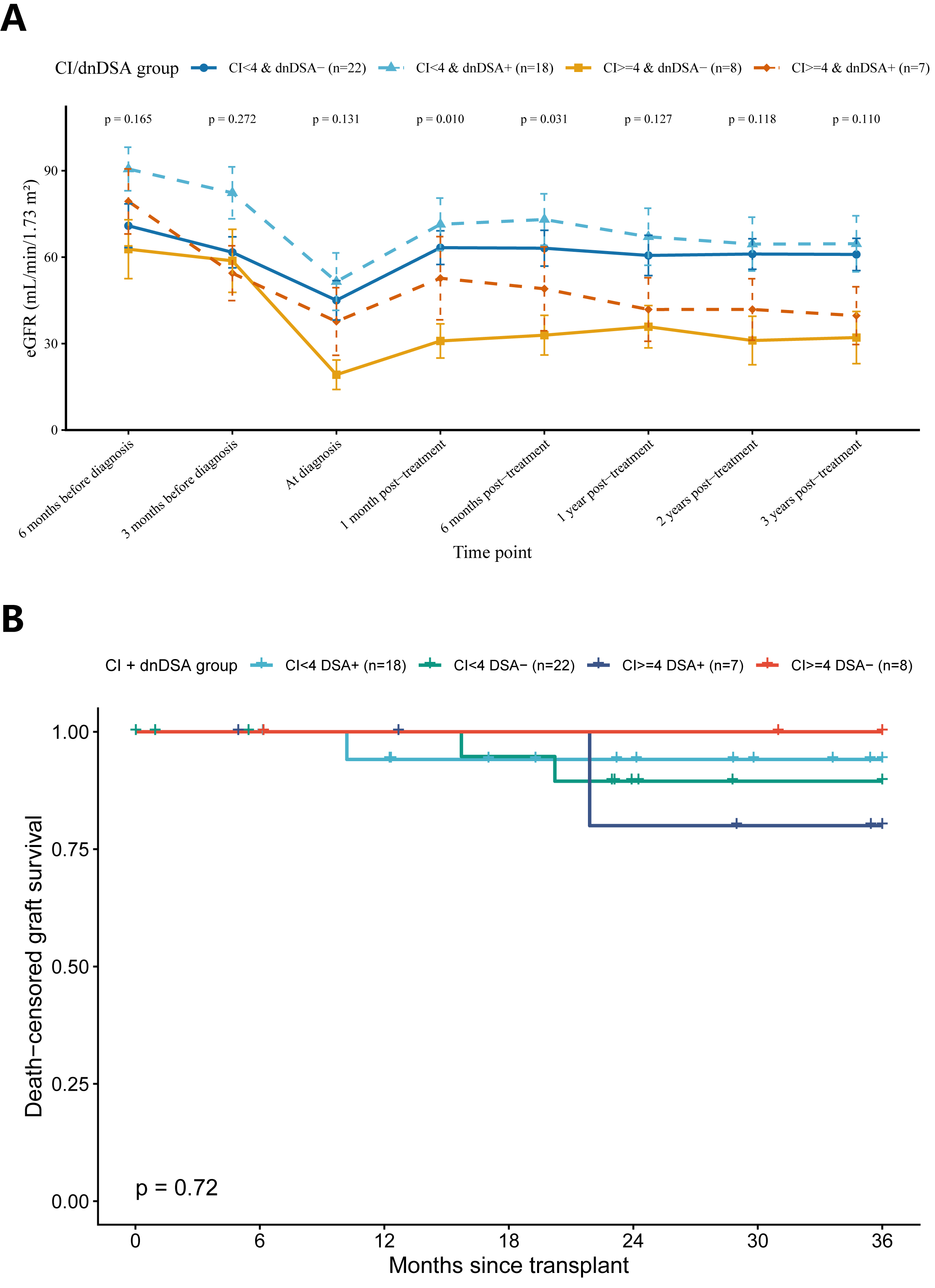


Figure.S5 Prognosis of graft stratified by combined CI score and dnDSA status.

A, eGFR levels at different time points in subgroups defined by CI score and dnDSA status. Blue curve: CI＜4&dnDSA-; Cyan curve: CI＜4&dnDSA+; Orange curve:CI≥4&dnDSA-; Red curve:CI≥4&dnDSA+. B, Kaplan-Meier plot in subgroups defined by CI score and dnDSA status. Cyan curve: CI＜4&dnDSA+; Green curve: CI＜4&dnDSA-; Purple curve: CI≥4&dnDSA+; red curve: CI≥4&dnDSA-.
